# PATTERN OF SEXUALLY TRANSMITTED INFECTIONS IN A TERTIARY CARE CENTRE AT PUDUCHERRY

**DOI:** 10.4103/0019-5154.57611

**Published:** 2009

**Authors:** S Abarna Devi, T P Vetrichevvel, Gajanan A Pise, Devinder Mohan Thappa

**Affiliations:** *From the Department of Dermatology and STD, Jawaharlal Institute of Postgraduate Medical Education and Research (JIPMER), Puducherry - 605 006, India.*

**Keywords:** *Herpes genitalis*, *HIV infection*, *pattern*, *sexually transmitted infections*

## Abstract

**Background:**

The prevalence of sexually transmitted infections (STI) varies widely from region to region in our country.

**Aims:**

To highlight the pattern of STIs and the profile of patients with HIV infection in STD patients as seen at our hospital.

**Methods:**

A retrospective chart analysis of clients attending STI clinic, JIPMER, Puducherry, from June 2004 to June 2006 was done.

**Results:**

A total of 866 clients attended our STI clinic, out of whom 435 (50.2%) had proven STI. STIs were more common in men, with a male (290): female (145) ratio of 2:1. Their age ranged from 1 year to 75 years (mean age = 32.38 years) with the maximum number of patients in the age group of 21-30 years, while children constituted only 2.8%. Herpes genitalis (107 patients, 32.8%) was the most common ulcerative STI, while genital wart was the most common nonulcerative STI (56 patients, 17.1%). Non-gonococcal urethritis (46 patients, 14.1%) was more common than gonococcal urethritis. HIV infection was the most common STI in our study, at an alarmingly high rate of 34.5% (151/435). HIV seropositivity was more common in patients who presented with ulcerative STIs than with nonulcerative STIs.

**Conclusions:**

Herpes genitalis was the most common ulcerative STD, while genital wart was the most common nonulcerative STI in our study. The prevalence of HIV among STI clients in India has been on the rise, but has quite alarmingly become the most common STI in our study.

## Introduction

The prevalence of sexually transmitted infections (STI) varies widely from region to region in our country. A systematic, regional periodic synopsis of the prevalence of STIs among STI clinic attendees would not only help to study the changing trends of STIs, but also to assess the effectiveness of control programs. During the past decade, there is overwhelming evidence that both ulcerative and nonulcerative STIs promote HIV transmission by augmenting HIV infectiousness and susceptibility.[1] An increasing trend of HIV seropositivity among the STI patients prompted us to analyze this relation. This study highlights the pattern of STIs and the profile of patients with HIV infection in STI patients as seen at our hospital for a period of 2 years.

## Materials and Methods

A retrospective chart analysis of clients attending STI clinic, JIPMER, Puducherry, from June 2004 to June 2006 was done. The diagnosis was based on clinical history, examination, and relevant laboratory investigations. HIV antibody testing and VDRL test were done in all patients after due consent.

## Results

A total of 866 clients had attended our STI clinic, out of whom 435 (50.2%) had proven STI. A majority of them (355 patients, 81.6%) were from the adjoining areas of Tamil Nadu, while 71 patients (16.3%) were from Puducherry. STIs were more common in men, with a male (290): female (145) ratio of 2:1. Their age ranged from 1 year to 75 years (mean age = 32.38 years) with the maximum number of patients in the age group of 21-30 years [Table 1], while children constituted only 2.8%. The predominant mode of contact was heterosexual (397 patients, 89.6%) followed by bisexual (20 patients, 4.6%), and homosexual (7 patients, 1.6%). A history of unprotected sexual intercourse with a commercial sex worker (CSW) was elicited in 69.7% of the patients. Only 8.34% of STI patients had reported consistent use of condoms during their non-marital sexual encounters. Herpes genitalis (107 patients, 32.8%) was the most common ulcerative STI, while genital wart was the most common nonulcerative STI (56 patients, 17.1%). Non-gonococcal urethritis (46 patients, 14.1%) was more common than gonococcal urethritis [Table 2].

**Table 1:** Age-wise distribution of STI patients

Age	Sex		Total (%)
		
	Male (%)	Female (%)	
0-10	7 (1.6)	3 (0.7)	10 (2.3)
11-20	16 (3.7)	10 (2.3)	26 (6.0)
21-30	107 (24.6)	64 (14.7)	171 (39.3)
31-40	90 (20.7)	50 (11.5)	140 (32.2)
41-50	46 (10.6)	17 (3.9)	63 (14.5)
51-60	18 (4.1)	1 (0.2)	19 (4.4)
61-70	4 (0.9)	0	4 (0.9)
71-80	2 (0.5)	0	2 (0.5)
Total	290 (66.7)	145 (33.3)	435 (100)

STI = Sexually transmitted infections

**Table 2:** Profile of STD patients

Diagnosis	Sex		Total (%)
		
	Male (%)	Female (%)	
Ulcerative			
Herpes genitalis	80 (24.5)	27 (8.9)	107 (32.8)
Syphilis	24 (7.3)	14 (4.3)	38 (11.6)
LGV	3 (0.9)	3 (0.9)	6 (1.8)
Chancroid	0	5 (1.8)	5 (1.8)
Non-specific ulcer	14 (4.3)	5 (1.8)	19 (5.82)
Non-ulcerative			
Genital warts	36 (11)	20 (6.1)	56 (17.1)
Genital molluscum contagiosum	6 (1.8)	3 (0.9)	9 (2.76)
Urethritis			
Gonococcal	6 (1.8)	0	6 (1.8)
Non-gonococcal (NGU)	46 (14.1)	0	46 (14.1)
Others			
Vulvo vaginal	0	22 (6.7)	22 (6.7)
candidiasis (VVC)			
Bacterial vaginosis	0	12 (3.68)	12 (3.68)
Total	215 (65.95)	111 (34.04)	326 (100)

HIV infection was the most common STI in our study, at an alarmingly high rate of 34.5% (151/435). A majority of these cases were in the age group of 21-30 years (39.1%) with a male: female ratio of 1.6: 1. Among patients with HIV seropositivity, the predominant mode of contact was heterosexual (138 patients, 91.4%), followed by bisexual (5 patients, 3.6%), and homosexual (2 patients, 1.2%). Seventy six percent of the HIV positive men had a history of contact with a CSW. HIV seropositivity was more common in patients who presented with ulcerative STIs than with nonulcerative STIs [Table 3]. Other symptomatic presentations of HIV seropositive patients included extensive oral candiasis (14 patients), scabies (8), constitutional symptoms (8), pruritic papular dermatosis (8), multidermatomal herpes zoster (5), extensive tinea corporis (5), and others (11). HIV seropositivity among STI clients presenting for voluntary testing (asymptomatic at presentation) was 21.7%, which formed one-third of our HIV patients.

**Table 3:** Profile of HIV among STD patients

Concomitant STD	No. of patients (%)
Ulcerative	
Herpes genitalis	13 (8.6)
Syphilis	7 (4.63)
Chancroid	2 (1.32)
Herpes genitalis+chancroid	1 (0.66)
Others	
Genital warts	11 (7.28)
Gonorrhea	1 (0.66)
Non-gonococcal urethritis	3 (1.98)
Vulvo vaginal candidiasis	4 (2.64)

## Discussion

Nearly half of our study population had one diagnosed STI. Although the epidemiology of STIs is male dominated, there is a gradual trend toward an increase in female attendees in STI clinic as evident in previous studies.[2,3] Even in our institute this changing proportion is reflected in the male: female ratio dropping from 3.7:1 in 1984 to 2:1 in the present study.[4] This could be attributed to effective contact tracing, spouse screening, and improved health care-seeking behavior in women, thanks to increasing female literacy rate.

STIs among teenagers have been reported to be as high as 24% by Chaudary *et al*. Arora *et al.,* and Singh *et al.*, but studies from our institute have mostly reported a lesser prevalence (6-12%), including the present study.[4–6] In our study, it was found that the majority of cases were in the peak reproductive age group of 21-30 years, which was in concordance with most other studies.[2,4–6]

HIV was the most common STI seen in our study (34.7%). Similar to a previous study from this institute, herpes genitalis was the most common ulcerative STI (32.8%) and continues to increase in frequency when compared to earlier studies.[4,7] Similar higher incidence of herpes genitalis has been reported by studies by Parmar *et al.* (27.9%) and Kumar *et al.* (19.7%).[2,8] A low prevalence of the herpes genitalis has been reported by Chopra *et al.* (9.4%) and Manas *et al.* (6.8%).[8,9]

Although syphilis was the second most common ulcerative STID, its incidence had decreased when compared to previous years[4,7] and latent syphilis was the most common presentation (78.6%). Manas *et al.* (15.6%) amd Parmar *et al.* (28.1%) have shown a higher incidence.[8,9] Chancroid seems to be on the decline as evident from other studies as well.[4,7,8]

Genital warts showed a high incidence (17.6%) similar to studies by Kumar *et al*. (25.2%), Gupta *et al.* (18.11%), and Aggarwal *et al.* (19.35%) with an increasing trend over the years.[2,3,7] Our study showed a relatively higher incidence of non-gonococal urethritis(14.1%) than gonnococal urethritis (1.8%), while most other studies have shown the contrary.[2,5,8] This general trend of decrease in bacterial STIs could be attributed to the ‘Syndromic treatment’ of STIs by peripheral health workers and private practitioners along with widespread use of broad spectrum antibiotics for other illnesses. Viral infections tend to persist or recur necessitating repeated consultations. The recurrent and unremitting symptoms of viral STIs coupled with media propaganda of HIV and AIDS prompts these patients to report to a higher centre for treatment and voluntary testing to rule out HIV disease.

The prevalence of HIV among STI clients in India has been on the rise[7,9,10] but has quite alarmingly become the most common STI in our study (34.7%). Only a very few studies from Bombay and Pune[10,11] had shown a similar high prevalence. The proportion of female clients is also increasing[7,12,13] (present study 1.6:1) with their maximum prevalence (30 patients, 52.6%) in the peak reproductive age group of 21-30 years suggesting that the infection is spreading from high-risk population to low-risk population. This might lead to increased perinatal transmission and more HIV-positive children. HIV seropositivity was more commonly seen with ulcerative STIs, especially herpes genitalis, closely followed by genital warts, features consistent with the general trends noted with most other studies.[7,12]

The increasing media propaganda and health education about the transmission of HIV disease and the need to test for the same if they develop particular symptoms have brought more asymptomatic patients for voluntary screening, but the continuing aberrant sexual behavior and low condom usage show that the prevention arm of HIV and STD control is not yet fully functional.
